# Diagnostic Power of Galectin-3 in Rheumatic Diseases

**DOI:** 10.3390/jcm9103312

**Published:** 2020-10-15

**Authors:** Ewa Gruszewska, Bogdan Cylwik, Ewa Gińdzieńska-Sieśkiewicz, Otylia Kowal-Bielecka, Barbara Mroczko, Lech Chrostek

**Affiliations:** 1 Department of Biochemical Diagnostics, Medical University of Bialystok, Waszyngtona St. 15A, 15-269 Bialystok, Poland; mroczko@umb.edu.pl (B.M.); chrostek@umb.edu.pl (L.C.); 2 Department of Pediatric Laboratory Diagnostics, Medical University of Bialystok, Waszyngtona St. 17, 15-274 Bialystok, Poland; cylwikb@umb.edu.pl; 3 Department of Rheumatology and Internal Diseases, Medical University of Bialystok, Sklodowskiej-Curie 24A, 15-276 Bialystok, Poland; ewa.gindzienska-sieskiewicz@umb.edu.pl (E.G.-S.); otylia@umb.edu.pl (O.K.-B.); 4 Department of Neurodegeneration Diagnostics, Medical University of Bialystok, Waszyngtona St. 15A, 15-269 Bialystok, Poland

**Keywords:** galectin-3, rheumatoid arthritis, systemic sclerosis, systemic lupus erythematosus

## Abstract

Background: The purpose of our study was to assess the diagnostic power of galectin-3 and compare its between rheumatic diseases and with routinely used tests such as CRP and ESR. Methods: Eighty-two patients with rheumatoid arthritis (RA), 49 patients with systemic sclerosis (SSc), and 18 patients with systemic lupus erythematosus (SLE) were enrolled in this study. The control group comprised 30 healthy controls. Serum galectin-3 concentration was measured using immunochemical method. Results: The galectin-3 concentration were significantly elevated in the RA, SSc, and SLE in comparison to the controls (*p* = 0.000, *p* = 0.000, *p* < 0.001; respectively). However, there were no significant differences in the serum galectin-3 levels between rheumatic diseases (H = 0.395, *p* = 0.821). In RA and SSc patients, galectin-3 positively correlated with erythrocyte sedimentation rate (R = 0.332, *p* = 0.004; R = 0.384, *p* = 0.009; respectively). ROC analysis revealed that galectin-3 had an excellent diagnostic power in RA (AUC = 0.911) and SSc (AUC = 0.903) and very good for SLE (AUC = 0.859). Conclusion: We concluded that diagnostic power of serum galectin-3 is as great as CRP and ESR in rheumatic diseases and it can be a very good laboratory marker in RA and SSc patients and a useful tool in the diagnosis of SLE.

## 1. Introduction

Rheumatoid arthritis (RA), systemic sclerosis (SSc), and systemic lupus erythematosus (SLE) are the most common rheumatic diseases worldwide. All of them are chronic systemic autoimmune diseases characterized by inflammation and hyperplasia, but the pathogenesis of these diseases is still not fully understood [1,2,3]. Previous studies have shown that rheumatic diseases are accompanied by a chronic inflammatory process, which is most often manifested by changes in the concentration of acute phase proteins [4]. Consequently, an advanced inflammatory response promotes the development of intense fibrosis and accumulation of extracellular matrix molecules (e.g., chemokines, cytokines, growth factors, proteins) [5,6].

Galectin-3 is a member of the β-galactoside-binding lectins family [7,8,9]. The galectin-3 molecule has a domain structure. There are two structural domains: the N-terminal domain containing the phosphorylation site and the C-terminal domain with the carbohydrate recognition domain—CRD. In human genome, this lectin is coded by a single gene LGALS3 situated on chromosome 14. Galectin-3 is predominantly located in the cytoplasm, but it has also been detected in the nucleus of cell, what suggesting a multifunctionality of this protein. Consequently, galectin-3 is involved in many biological processes, such as cell and cell recognition, cell and extracellular matrix adhesion, cell growth and differentiation, cell cycle and signaling, apoptosis, and angiogenesis. Besides, galectin-3 is widely expressed protein in human cells and tissues [8,9,10]. Expression of galectin-3 has been shown in many types of cells (e.g., neutrophils, monocytes, macrophages, dendritic cells, mast cells, osteoclasts, fibroblasts, cancer cells), and what is most importantly—in all types of immune cells. The presence of galectin-3 has been demonstrated also in the tissues of the lungs, spleen, stomach, and also in the heart, kidneys, pancreas, and liver. Thus, a significant increase of galectin-3 level in the blood serum is observed in many pathological processes which take place in various tissues. Previous studies have already shown that galectin-3 plays an important role in the development of liver or pulmonary fibrosis [9,11,12].

Taking into account the presence of galectin-3 in immune cells, and the role of immune system in rheumatic diseases the purpose of our study was to assess the serum galectin-3 concentration and compare its diagnostic values with the routinely used tests between rheumatic diseases, including rheumatoid arthritis, systemic sclerosis, and systemic lupus erythematosus.

## 2. Materials and Methods

### 2.1. Patients

The study group consisted of 149 patients with rheumatic diseases (124 females and 25 males), aged 19–85 years (median age: 51 years) admitted to the Department of Rheumatology and Internal Diseases, Medical University of Bialystok. The patients were divided into subgroups according to the diagnosis of rheumatic diseases: rheumatoid arthritis (RA)—82 patients (69 females and 13 males), systemic sclerosis (SSc)—49 patients (39 females and 10 males), and systemic lupus erythematosus (SLE)—18 patients (16 females and 2 males). The diagnosis of RA was confirmed according to the ACR 2010 classification criteria [13]. In these criteria, the diagnosis of RA is based on the presence of synovitis in at least one joint, and achievement of a total score of ≥6 from the individual scores in four domains. RA activity was evaluated by disease activity score (DAS28) calculated by a following complex formula which included the number of tender (t28) and swollen (s28) joints, erythrocyte sedimentation rate (ESR), and visual analog scale (VAS)

DAS28 = 0.56 × sqrt(t28) + 0.28 × sqrt(s28) + 0.7 × ln(ESR) + 0.014 × VAS

DAS28 in RA patients ranges from 2.32 to 8.0 (median: 6.0). The recognition of systemic sclerosis was made on the ACR/EULAR 2013 classification criteria [14]. According to them, patients with a total score of ≥9 are classified as having definite SSc. In turn, the diagnosis of systemic lupus erythematosus was based on the SLICC 2012 classification criteria [15]. These require fulfillment of at least four of the criteria, including at least one clinical and one immunologic criterion. To eliminate of an effect of cardiomyopathy on the level of galectin-3, all patients had to carry out the heart ultrasonography.

The control group consisted of 30 healthy subjects recruited from hospital workers (19 females and 11 males) aged 21–54 years (median age: 25 years). Informed consent was obtained from all individual participants (healthy and sick) included in the study. This study was in accordance with Helsinki declaration and was approved by the Bioethical Committee working at the Medical University of Bialystok (approval no. R-I-002/416/2018).

### 2.2. Blood Sampling

Blood samples from patients with rheumatic diseases and healthy subjects were collected by peripheral vein puncture. The sera were separated by centrifugation at 1500× *g* for 10 min at room temperature and stored at −86 °C until assayed. Besides serum, a part of each blood samples was collected into tubes containing liquid sodium citrate for determination of ESR and EDTA-2 for hematological analysis.

### 2.3. Laboratory Assessments

Galectin-3 concentration was measured by the chemiluminescent microparticle immunoassay with Abbott reagents (Architect Galectin-3, Abbott, Germany) on the Architect ci8200 analyzer (Abbott Laboratories, Abbott Park, IL, USA).

Another biochemical assay such as CRP was measured by the immunoturbidimetric method on the Architect ci 8200 analyzer. ESR was assayed by Westergren method on the Sediplus S 2000 (Sarstedt, Germany). Hemoglobin level and PLT were determined by using routine methods on the Sysmex XS-800i analyzer (Sysmex Corporation, Singapore).

### 2.4. Statistical Analysis

Statistical analysis was performed using Statistica 13.1 PL (StatSoft, Poland). Because of abnormal distribution of tests, we used non-parametric tests. The results were given as medians and ranges. The differences between study and control groups were evaluated by Mann–Whitney *U* test. To test the hypothesis about the differences between rheumatic diseases, ANOVA rank Kruskal–Wallis test was done. The correlation between variables was assessed by Spearman’s rank correlation coefficient. The *p*-values of less than 0.05 were considered significant. To calculate the diagnostic accuracy of galectin-3 in rheumatic diseases the area under the receiver operating characteristic curve (AUROC) was calculated. Diagnostic sensitivity, specificity, accuracy, positive (PPV), and negative predictive values (NPV) were counted using a cut-off point as a 97.5th percentile of the control group.

## 3. Results

The demographic and laboratory data of all study groups (patients with rheumatic diseases and healthy subjects) are summarized in the Table 1. The median of serum galectin-3 concentration was significantly elevated in RA (18.75 ng/mL), SSc (19.4 ng/mL), and SLE (19.2 ng/mL) in comparison to the healthy subjects (9.45 ng/mL) (Z = 5.710, *p* = 0.000; Z = 5.323, *p* = 0.000; Z = 3.596, *p* < 0.001; respectively). The median levels of ESR, CRP, and PLT in RA, SSc, and SLE were also significantly increased compared to the control group (with exception of PLT level in SLE), whereas the median of HGB was decreased in comparison to the controls (Table 1).

The analysis of variance revealed that rheumatic diseases did not affect the galectin-3 concentration (H = 0.395, *p* = 0.821), CRP (H = 4.798, *p* = 0.091), PLT (H = 5.061, *p* = 0.080), and HGB (H = 3.935, *p* = 0.140), but effect the ESR (H = 13.001, *p* = 0.002). In RA patients, galectin-3 concentration positively correlated with the age of patients (R = 0.327, *p* = 0.003), but there were no significant correlation between galectin-3 concentrations and DAS28 (R = 0.236, *p* = 0.060). Moreover, in RA and SSc patients, galectin-3 concentration positively correlated with ESR (R = 0.332, *p* = 0.004; R = 0.384, *p* = 0.009; respectively). In SLE patients, galectin-3 concentration did not correlate with indicators of inflammatory activity.

The diagnostic power of galectin-3 in rheumatic diseases (RA, SSc, and SLE) is presented in Table 2. The results of this study showed that galectin-3 had a high diagnostic specificity in all rheumatic diseases accompanied by diagnostic sensitivity placed between ESR and CRP values. The sensitivity of galectin-3 in SLE is a little lower but still have a high specificity (95%). The diagnostic accuracy (ACC) of galectin-3 was high and similar in all rheumatic diseases (about 80%). The positive predictive value (PPV) of galectin-3 was higher than PPV of CRP and ESR in all rheumatic diseases.

**Figure 1:**
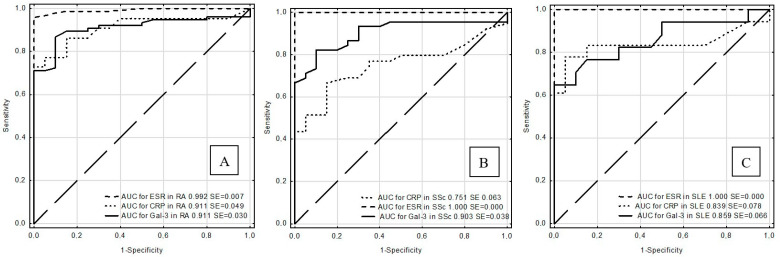
ROC curves for galectin-3, CRP, and ESR in rheumatoid arthritis (RA) (**A**), systemic sclerosis (SSc) (**B**), and systemic lupus erythematosus (SLE) (**C**).

The AUCs for Gal-3 and CRP were significantly different than AUC for ESR in RA (*p* < 0.001 for both comparison) and in SLE (*p* < 0.001, *p* = 0.033, respectively). In SSc, there were no significant differences between AUCs for Gal-3, CRP, and ESR (Figure 1).

**Figure 2:**
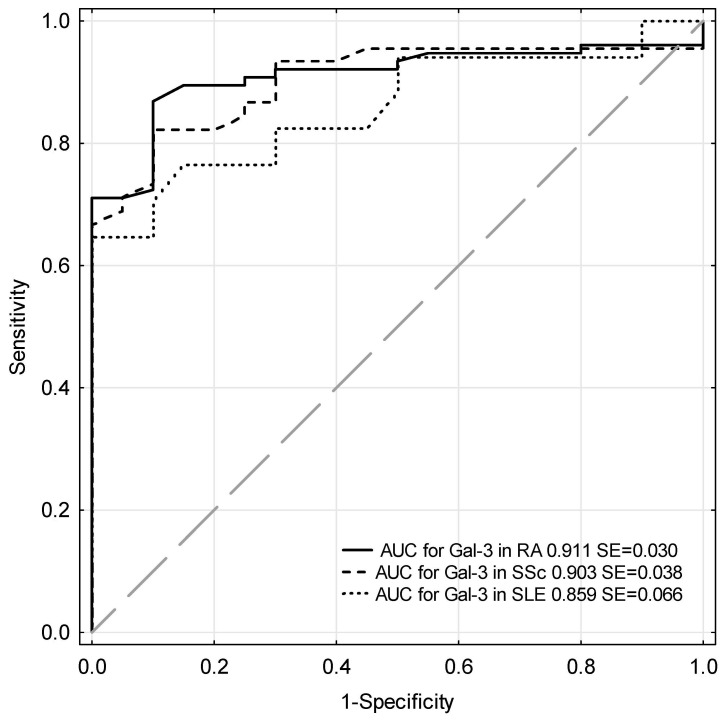
ROC curves for serum galectin-3 in rheumatic diseases.

ROC analysis revealed that galectin-3 had an excellent diagnostic power in RA and SSc and very good diagnostic power for SLE, because the area under curves (AUCs) were as follows in decreasing order: for RA—0.911, for SSc—0.903, and for SLE—0.859 (Figure 2).

## 4. Discussion

Throughout the past decade, galectin-3 has attracted the attention of researchers due to its regulatory role in immune response, inflammation, and fibrosis [9,16]. Thus, several studies already showed that galectin-3 plays an important role in the development of different pathological conditions. Some studies proved that increased galectin-3 concentration is associated with high heart failure risk [17]. There are evidences that galectin-3 concentration increases significantly, both in chronic and acute heart failure, according to the progression of disease [18,19]. In pulmonary and liver fibrosis, serum concentration of galectin-3 was found to be elevated [9,11,12,20]. Accordingly, mentioned studies proposed that determination of serum galectin-3 concentration could be a useful marker of active fibrosis in the course of these diseases. Similar studies suggested association of high serum galectin-3 levels with inflammatory disorders and autoimmune diseases, including rheumatic diseases [8,16]. The diagnosis of rheumatic conditions, especially in early stages of the disease is still very difficult. Therefore, in recent years, interest in finding useful circulating biomarkers for early diagnosis and which may reflect the progression of rheumatic diseases does not diminish.

Due to these facts, we try to assess the serum galectin-3 levels, its diagnostic values and potential association with progression of disease in the most common rheumatic diseases. The results of this study have shown increased serum concentration of galectin-3 in all studied rheumatic diseases: rheumatoid arthritis, systemic sclerosis, and systemic lupus erythematosus in comparison to the healthy subjects. The results of our study were comparable with those reported in literature. Previous studies documented the changed levels of galectin-3 in patients with RA, juvenile idiopathic arthritis (JIA), SSc, ankylosing spondylitis (AS), or Behcet’s diseases (BD) [6,21,22,23,24]. For example, Ohshima et al. have demonstrated increased levels of galectin-3 both in RA synovial fluid and serum in comparison to patients with osteoarthritis and healthy subjects [6]. Similar results were obtained by Ezzat et al. in JIA patients [21]. Koca et al. also reported higher serum galectin-3 concentration in systemic sclerosis and Cao et al. found similar higher serum galectin-3 concentration in patients with ankylosing spondylitis [22,23]. Lee et al. showed a higher serum galectin-3 concentration in patients with Behcet’s disease than the healthy ones, and that the active BD patients had higher galectin-3 levels than these with the inactive BD. While the levels of galectin-3 binding protein were not different between BD patients and controls, but it was higher in active BD than in non-active BD [24]. The mechanism by which galectin-3 is released into the extracellular space is still not fully understood. There are findings which suggested that galectin-3 molecule on its own has the capacity to traverse the lipid bilayer [25]. Galectin-3 has a direct effect on immune system and inflammatory responses by modulating cell adhesion of various type of immune cell. The most probable mechanism is that, upon tissue injury, galectin-3 which is normally stored in the cytoplasm is actively secreted to the blood and other biological fluids by activated and damaged cells. Therefore, inflammation and dysfunction of immune response may increase serum galectin-3 concentration in patients with different rheumatic diseases. There is well known that galectin-3 plays an important role in the development of inflammation by interacting with various cytokines and chemokines. Moreover, galectin-3 has been suggested to play a key role in inducing fibrosis in different tissues [9,11,12]. The effects of galectin-3 clearly depend on cellular or tissue localization.

In the most of diseases mentioned above the concentrations of galectin-3 were significantly associated with the C-reactive protein (CRP) and disease activity scores. It is suggested that determination of serum galectin-3 may be utilized marker for the disease’s prognosis. Although the increased level of galectin-3 is not specific for example in RA patients, as showed by Ohshima et al. [6]. Also, in our study we did not report a significant correlation between galectin-3 concentration and CRP values, also there was no correlation with DAS28. On the other hand, we observed a positive correlation of galectin-3 with ESR in RA and SSc patients. Moreover, we noted a positive correlation with age of RA patients.

While increased serum levels of galectin-3 have been previously reported in patients with rheumatic diseases, this is probably the first study investigating the diagnostic values of serum galectin-3 in the diagnosis of selected rheumatic diseases. Our study showed that galectin-3 has a high diagnostic sensitivity, specificity, PPV, and diagnostic accuracy in rheumatoid arthritis and systemic sclerosis patients. For the SLE patients the sensitivity was a little lower than in RA and SSc patients. Besides, galectin-3 in SLE patients has high specificity and PPV values. It is a consequence of the small number of false positive results. Moreover, we showed that galectin-3 had a very high diagnostic power (area under the ROC curve) for all tested rheumatic diseases, making galectin-3 a good diagnostic marker. Diagnostic power was excellent for RA and SSc patients (ACC over 0.9) and very good for SLE patients (ACC over 0.8).

## 5. Conclusions

In conclusion, we demonstrated that serum galectin-3 concentration is elevated in RA, SSc, and SLE in comparison with healthy subjects. Therefore, the results obtained in our study suggest that determination of serum galectin-3 concentration may be a useful tool in the diagnosis and treatment of these diseases. Moreover, galectin-3 due to the high diagnostic power can be a valuable surrogate marker for the diagnosis of rheumatic diseases, particularly RA and SSc.

## Figures and Tables

**Table 1:** Demographic and laboratory data of rheumatic patients and control group

	Age (Years)	Disease Duration	Galectin-3 (ng/mL)	ESR (mm/h)	CRP (mg/L)	PLT (10^9^/L)	HGB (g/dL)
RA *n* = 82	58.5 20–85	9 years 2 months–35 years	18.75 3.8–50.3 *p* = 0.000 *	47.5 6–120 *p* = 0.000 * p = 0.002 ^#^	6.4 0.3–151.9 *p* < 0.001 *	289 24–839 *p* = 0.020 *	11.65 7.9–15.2 *p* < 0.000 *
SSc *n* = 49	52 19–77	6.5 years 3 months–30 years	19.4 2.5–64.7 *p* = 0.000 *	23 2–100 *p* = 0.000 *	2.3 0.2–306.1 *p* = 0.002 *	279 193–398 *p* = 0.008 *	12.35 8.5–14.8 *p* < 0.001 *
SLE *n* = 18	38 23–70	9.7 years 1 year–21 years	19.2 7.5–117.6 *p* < 0.001 *	30 10–100 *p* = 0.000 *	3.85 0.2–56.5 *p* < 0.001 *	228 97–588 *p* = 0.822	12.3 8.8–13.6 *p* < 0.001 *
Control group *n* = 30	25 21–54	-	9.45 7.3–15	5.5 4–8	0.65 0.3–3.2	235 166–386	13.7 11.3–15.5

Data are median and ranges. Significant differences at *p* < 0.05. The differences between tested groups and controls were estimated by Mann–Whitney *U* test. The differences between rheumatic subgroups estimated by ANOVA rank Kruskal–Wallis test. *—when comparing tested groups and controls ^#^—when comparing RA and SSc. RA—rheumatoid arthritis; SSc—systemic sclerosis; SLE—systemic lupus erythematosus; ESR—erythrocyte sedimentation rate; CRP—C-reactive protein; PLT—platelet; HGB—hemoglobin.

**Table 2:** Diagnostic power of galectin-3, ESR, and CRP in rheumatic diseases

Rheumatic Disease	Test	Cut-off (ng/mL)	Sensitivity (%)	Specificity (%)	ACC (%)	PPV (%)	NPV (%)	AUC ± SE
RA	Gal-3	15	71.1	95	76	98.2	46.3	0.911 ± 0.030
	ESR	8	98.6	85	95.7	95.9	94.4	0.992 ± 0.007
	CRP	3.2	72.7	95	83.3	94.1	76	0.911 ± 0.049
SSc	Gal-3	15	68.9	95	76.9	96.9	57.6	0.903 ± 0.038
	ESR	8	100	85	88.9	70	100	1.000 ± 0.000
	CRP	3.2	43.6	95	61	94.4	46.3	0.751 ± 0.063
SLE	Gal-3	15	64.7	95	81.1	91.7	76	0.859 ± 0.066
	ESR	8	100	85	92.3	86.4	100	1.000 ± 0.000
	CRP	3.2	61.1	95	78.9	91.7	73.1	0.839 ± 0.078

RA—rheumatoid arthritis; SSc—systemic sclerosis; SLE—systemic lupus erythematosus; ACC—diagnostic accuracy; PPV—positive predictive value; NPV—negative predictive value; AUC—area under ROC curve; SE—standard.

## References

[1] Calabresi E., Petrelli F., Bonifacio A.F., Puxeddu I., Alunno A. (2018). One year in review 2018: Pathogenesis of rheumatoid arthritis. Clin. Exp. Rheumatol..

[2] Stern E.P., Denton C.P. (2015). The pathogenesis of systemic sclerosis. Rheum Dis Clin. North. Am..

[3] Zucchi D., Elefante E., Calabresi E., Signorini V., Bortoluzzi A., Tani C. (2019). One year in review 2019: Systemic lupus erythematosus. Clin. Exp. Rheumatol..

[4] Saroha A., Biswas S., Chatterjee B.P., Das H.R. (2011). Altered glycosylation and expression of plasma alpha-1-acid glycoprotein and haptoglobin in rheumatoid arthritis. J. Chromatogr. B Anal. Technol. Biomed. Life Sci..

[5] LeRoy E.C., Black C., Fleischmajer R., Jablonska S., Krieg T., Medsger T.A., Rowell N., Wollheim F. (1988). Scleroderma (systemic sclerosis): Classification, subsets and pathogenesis. J. Rheumatol..

[6] Ohshima S., Kuchen S., Seemayer C.A., Kyburz D., Hirt A., Klinzing S., Michel B.A., Gay R.E., Liu F.T., Gay S. (2003). Galectin-3 and its binding protein in rheumatoid arthritis. Arthritis Rheum..

[7] Henderson N.C., Seith T. (2009). The regulation of inflammation by galectin-3. Immunol. Rev..

[8] Chen H.Y., Liu F.T., Yang R.Y. (2005). Roles of galectin-3 in immune responses. Arch. Immunol. Ther. Exp..

[9] Li L.C., Li J., Gao J. (2014). Functions of galectin-3 and its role in fibrotic diseases. J. Pharm. Exp. Ther..

[10] Dhirapong A., Lleo A., Leung P., Gershwin M.E., Liu F.T. (2009). The immunological potential of galectin-1 and -3. Autoimmun. Rev..

[11] Nishi Y., Sano H., Kawashima T., Okada T., Kuroda T., Kikkawa K., Kawashima S., Tanabe M., Goto T., Matsuzawa Y. (2007). Role of galectin-3 in human pulmonary fibrosis. Allergol. Int..

[12] Henderson N.C., Mackinnon A.C., Farnworth S.L., Poirier F., Russo F.P., Iredale J.P., Haslett C., Simpson K.J., Sethi T. (2006). Galectin-3 regulates myofibroblast activation and hepatic fibrosis. Proc. Natl. Acad. Sci. USA.

[13] Aletaha D., Neogi T., Silman A.J., Funovits J., Felson D.T., Bingham C.O., Birnbaum N.S., Burmester G.R., Bykerk V.P., Cohen M.D. (2010). Rheumatoid arthritis classification criteria: An American College of Rheumatology/European League against Rheumatism Collaborative Initiative. Arthritis Rheum..

[14] Van den Hoogen F., Khanna D., Fransen J., Johnson S.R., Baron M., Tyndall A., Matucci-Cerinic M., Naden R.P., Medsger T.A., Carreira P.E. (2013). Classification criteria for systemic sclerosis: An American College of Rheumatology/European League against Rheumatism Collaborative Initiative. Arthritis Rheum..

[15] Petri M., Orbai A.M., Alarcon G.S., Gordon C., Merrill J.T., Fortin P.R., Bruce I.N., Isenberg D., Wallace D.J., Nived O. (2012). Derivation and validation of the systemic lupus international collaborating clinics classification criteria for systemic lupus erythematosus. Arthritis Rheum..

[16] De Oliveria F.L., Gatto M., Bassi N., Luisetto R., Ghirardello A., Punzi L., Doria A. (2015). Galectin-3 in autoimmunity and autoimmune diseases. Exp. Biol. Med..

[17] McCullough P.A. (2014). Practical experience using galectin-3 in heart failure. Clin. Chem. Lab. Med..

[18] De Boer R.A., Yu L., Van Veldhuisen D.J. (2010). Galectin-3 in cardiac remodeling and heart failure. Curr. Heart Fail. Rep..

[19] Hrynchyshyn N., Jourdain P., Desnos M., Diebold B., Funck F. (2013). Galectin-3: A new biomarker for the diagnosis, analysis and prognosis of acute and chronic heart failure. Arch. Cardiovasc. Dis..

[20] Mackinnon A.C., Gibbons M.A., Farnworth S.L., Leffler H., Nilsson U.J., Delaine T., Simpson A.J., Forbes S.J., Hirani N., Gauldie J. (2012). Regulation of transforming growth factor-β1-driven lung fibrosis by galectin-3. Am. J. Respir. Crit. Care Med..

[21] Ezzat M.H., El-Gammasy T.M., Shaheen K.Y., Osman A.O. (2011). Elevated production of Gal-3 is correlated with juvenile idiopathic arthritis disease activity, severity, and progression. Int. J. Rheum. Dis..

[22] Koca S.S., Akbas F., Ozgen M., Yolbas S., Ilhan N., Gundogdu B., Isik A. (2014). Serum Gal-3 level in systemic sclerosis. Clin. Rheumatol..

[23] Cao M.-Y., Wang J., Gao X.-L., Hu Y.-B. (2019). Serum galectin-3 concentrations in patients with ankylosing spondylitis. J. Clin. Lab. Anal..

[24] Lee Y.J., Kang S.W., Song J.K., Park J.J., Bae Y.D., Lee E.Y., Lee E.B., Song Y.W. (2007). Serum Gal-3 and Gal-3 binding protein levels in Behçet’s disease and their association with disease activity. Clin. Exp. Rheumatol..

[25] Lukyanov P., Furtak V., Ochieng J. (2005). Galectin-3 interacts with membrane lipids and penetrates the lipid bilayer. Biochem. Biophys. Res. Commun..

